# Quantitative Structure–Activity Relationship (QSAR) Modeling of Chiral CCR2 Antagonists with a Multidimensional Space of Novel Chirality Descriptors

**DOI:** 10.3390/molecules30020307

**Published:** 2025-01-14

**Authors:** Ramanathan Natarajan, Ganapathy S. Natarajan, Subhash C. Basak

**Affiliations:** 1 Department of Research and Development, Saranathan College of Engineering, Panjappur, Tiruchirappalli 620 012, Tamil Nadu, India; 2 Department of Mechanical Engineering and Industrial Engineering, University of Wisconsin-Platteville, Platteville, WI 53818, USA; natarajang@uwplatt.edu; 3 Independent Researcher, 1802 Stanford Avenue, Duluth, MN 55811, USA; sbasak@d.umn.edu

**Keywords:** quantitative chirality structure–activity relationship (QCSAR), QSAR, chirality, chirality indices, CCR2 antagonists

## Abstract

The development of chirality descriptors for quantitative chirality structure–activity relationship (QCSAR) modeling has always attracted attention, owing to the importance of chiral molecules in pharmaceutical, agriculture, food, and fragrance industries, and environmental toxicology. The utility of a multidimensional space of novel relative chirality indices (RCIs) in the QCSAR modeling of twenty CCR2 antagonists is reported upon in this paper. The numerical characterization of chirality by the RCI approach gives a large pool of chirality descriptors with different degrees of mutual correlation (the correlation coefficient among the computed descriptors varied from 0.02 to 0.99). In the present study, the final data set contains 198 chirality descriptors for each of the twenty CCR2 antagonist molecules, providing a multidimensional space for modeling. The data reduction using principal component analysis resulted in the extraction of eight principal components (PCs). The linear regression using the principal component scores (PCSs) resulted in a three-predictor prediction model with good statistics: R^2^ = 0.823; Adj R^2^ = 0.790. The regression models were rebuilt using the chirality descriptors (RCIs) that are most correlated with each of the scores (PCSs) of the three principal components. The R^2^ value for the regression models with three RCIs as the predictors is 0.742 and the five-fold cross validation, R_cv_^2^, is 0.839. The new chirality descriptors, namely, the RCIs calculated using a different weighting scheme, provide a multidimensional space of chirality descriptors for a set of chiral molecules, and such a multidimensional chirality space is a powerful tool to build quantitative chiral structure–activity relationship (QCSAR) models.

## 1. Introduction

The term chirality was introduced by Lord Kelvin to explain the relation between the optical isomers or enantiomers that differ in their three-dimensional disposition around an asymmetric carbon atom, which are related as the object and the non-superimposable mirror image. In chemistry, there is a solubility rule to explain the solubility of solutes and it states that “Like dissolves like,” which means polar solvents dissolve polar solutes and non-polar solvents dissolve non-polar solutes. The same is true for the chiral distinction. Only a chiral environment can distinguish chiral isomers. Biological systems are chiral in nature, owing to the homochirality of the building blocks of the macromolecules. Thus, chirality is the biosignature present in all biological macromolecules [1]. The homochirality that is prevailing among the biological molecules is considered to have a cosmic origin [2] in support of these propylene oxides, since a chiral molecule has been identified in interstellar space [3]. The biological systems are enantio-selective, and the enantio-distinction plays a vital role in drug action, i.e., pharmacology [4,5]. The chiral distinction or enantio-selectivity is not a phenomenon relevant only to the pharmaceutical industry [6] but extends to other areas such as the agriculture [7], fragrance, and food industries [8], and environmental toxicology [9].

The QSAR approach has been extensively used to model various physiochemical properties, biological activities, and toxicological end points [10,11,12,13,14,15,16,17,18]. In the QSAR approach for modeling and predicting the property/activity under consideration, computed molecular descriptors are preferred and have been successfully used over the years. The large set of molecular descriptors used in the QSAR approach under the name of topological indices are derived from their molecular graphs. In a molecular graph, the atoms form the vertices, and the bonds form the edges. The topological indices are therefore computed based on the connectivity of atoms in the molecular graphs. In the case of enantiomers, though they have the object and the non-superimposable mirror image relation, the molecular connectivity remains the same. That is, enantiomers have identical molecular graphs; therefore, *R*- and *S*- isomers will have the same numerical values for the molecular descriptors and fail to distinguish them. To overcome this limitation in modifications, which are generally regarded as chiral modifications, chiral corrections are applied in the computation of the topological indices. These modifications may be classified into two major types:

(1) including a chiral correction into the connectivity matrix and computing the chiral descriptors.
(2) applying a chiral correction factor to the topological indices computed based on a well-defined algorithm, such that new Chiral TI = conventional TI × correction factor.

The second approach modifies the existing topological index; therefore, they must be computed as the first step. The different methods on the numerical characterization of chirality have resulted in chirality descriptors that could distinguish enantiomers and diastereomers [19,20,21,22,23,24]. In most of these approaches, only one type of index is derived and of course they satisfy the primary objective in differentiating the enantiomers. The numerical characterization approach that provides just a pair of descriptors for a set of enantiomers has a limitation in its application to different biological responses for the same set of chiral molecules. The stereospecific recognition of the same set of molecules by two different receptors may be completely uncorrelated. The chiral descriptors might be successful in modeling one of these biological activities of a set of enantiomers but fail to model other properties related to a different receptor. In such a scenario, a single set of descriptors may be able to model one of the two responses. This was illustrated in the dopamine sigma receptor and D2 receptor affinities for seven pairs of 3HPPs, 3-(3-hydroxyphynyl)piperidine [19]. These two biological responses are mutually uncorrelated (correlation coefficient r = 0.195). When a single set of chiral descriptors for these seven pairs of enantiomers were used, it could model the sigma receptor affinities but failed in modeling the D2 receptor affinities. In order to handle such a situation, a family of chiral descriptors was proposed [19]. Among these descriptors, some were mutually perfectly correlated (r = 0.999), while some were uncorrelated (r < 0.1). This approach was like that of Kier and Hall [16,17,19] in extending Randić connectivity indices [20] to calculate a family of topological indices for a given set of molecules. Similar to Kier and Hall’s approach, this approach gave the Randić connectivity index new dimensions and extended its applicability. The QSAR approach, which is a new approach of calculating a large pool of chirality indices for chiral molecules, was expected to have wider applicability to many different properties, because the chiral descriptors provide a multidimensional space. The new type of chirality indices proposed to differentiate enantiomers and diastereomers were called the relative chirality indices (RCIs).

**Figure 1:**
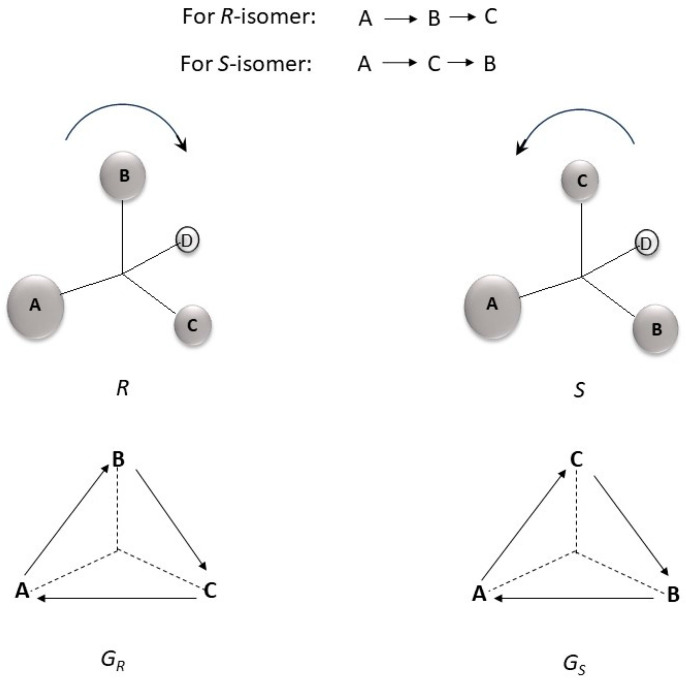
3D structure to 2D directed graph.

Readers may wonder how chirality, an intrinsically three-dimensional (3D) phenomenon, is characterized by a graph theoretic approach which is only two-dimensional (2D). Our approach of computing relative chirality indices may be looked upon as a method of transforming the 3D disposition of the four substituents attached to a chiral center into two directed graphs. The transition of CIP rules of ordering the four substituents attached to a chiral carbon into two directed graphs to represent *R*- and *S*- isomers is outlined in Figure 1. The four groups A, B, C, and D are attached to the chiral carbon, and the order of priority according to Cahn–Ingold–Prolog (CIP) rules is A > B > C > D. While assigning the R/S configuration, group D (least priority) is kept away from the viewer and the other three groups are placed in the front (Figure 1). This disposition is transferred into two directed graphs:

The two directed graphs differ in their adjacency matrix. The adjacency matrices for the two directed graphs are as follows:

$$\mathrm{Adjacency}\;\mathrm{matrix}\;\mathrm{for}\;\mathrm{the}\;R\;\mathrm{isomer}\;A(G_{R})=\left[\begin{array}{ccc} 0 & 1 & 0\\ 0 & 0 & 1\\ 1 & 0 & 0 \end{array}\right]$$

$$\mathrm{Adjacency}\;\mathrm{matrix}\;\mathrm{for}\;\mathrm{the}\;S\mathrm{isomer}\;A\left(G_{S}\right)=\left[\begin{array}{ccc} 0 & 0 & 1\\ 1 & 0 & 0\\ 0 & 1 & 0 \end{array}\right]$$

In order to derive indices from the directed graph, a set of formulas were proposed to calculate chirality indices that could distinguish the enantiomers. The initial formulation [25] was recently modified [22] to compute a pack of several chirality indices for a given set of enantiomers. The formulas to calculate the chirality descriptors, relative chirality indices (RCI), *^R^*RCI, and *^S^*RCI are given below:

(1) RRCI=δa+δa+δa×δb+δa+δa×δb+δa×δb×δc+δa×δb×δc×δd

(2) RSCI=δa+δa+δa×δc+δa+δa×δc+δa×δb×δc+δa×δb×δc×δd

where δ_a_, δ_b_, δ_c_, and δ_d_ are the weights assigned to the four substituents A, B, C, and D attached to the chiral carbon, while the CIP priority order is A > B > C > D.

This new approach enables the extension the of computation of RCIs to molecules with more than one chiral center. When a molecule has more than one chiral center, the RCI is computed for each center based on substituents attached, and the individual RCI values thus computed for each chiral center in a molecule is combined by taking a root mean square value to derive a single RCI for the chiral molecule (Equation (3)). Thus, RCIs can be calculated not only for enantiomers but also for diastereomers.

(3) $$\mathrm{RCI}=\sqrt{\frac{1}{N}}\sum_{i=1}^{N}{\mathrm{RCI}}_{i}^{2}$$

The calculation of the RCI and its application to model the biological activity of diastereomers was illustrated for the differential repellency (mosquito) of *SS* 220 [21], and recently, Natarajan et al. [19] extended the application of the large pool of RCIs calculated to model two biological responses for a set of seven pairs of enantiomers of 3-(3-hydroxyphenyl)piperidines. The present paper is an extension of this approach to model the biological activities of a diverse data set of chiral molecules.

Physiological and pathological processes in the human body are regulated by a system of chemokine and chemokine receptors, a subfamily of human Class A G-protein coupled receptors (GPCRs) [22,23]. The chemokine receptors play a significant role in the migration and localization of leukocytes. To date, the protein data bank (PDB) has a repository of the structures of 22 chemokine receptors. Urvas and Kellenberger [24] analyzed and compared their structures in a recent review. Out of the chemokine receptors, the CC-chemokine receptor 2 (CCR2) is the second most studied receptor. CCR2 is involved in various neurodegenerative disorders including Alzheimer’s disease, multiple sclerosis, and ischemic brain injury [25,26,27,28]. During the SARS-CoV-2 pandemic, the involvement of CCR2 in fighting the inflammation of lungs was extensively studied [29,30]. Hence, CCR2 has attracted attention as a therapeutic target for autoimmune diseases such as rheumatoid arthritis [27], cancer [25,29], traumatic brain injury [31], etc. In most of these, the overexpression of CCR2 is the main cause; therefore, the suppression or dampening by CCR2 antagonists is one of the therapeutic strategies. Owing to the therapeutic importance of CCR2, we considered developing QSAR models for CCR2 antagonists that are chiral using a large pool of RCIs computed based on various algorithms to assign weights to the four substituents attached to the chiral carbon.

## 2. Materials and Methods

### 2.1. Biological Data

**Figure 2:**
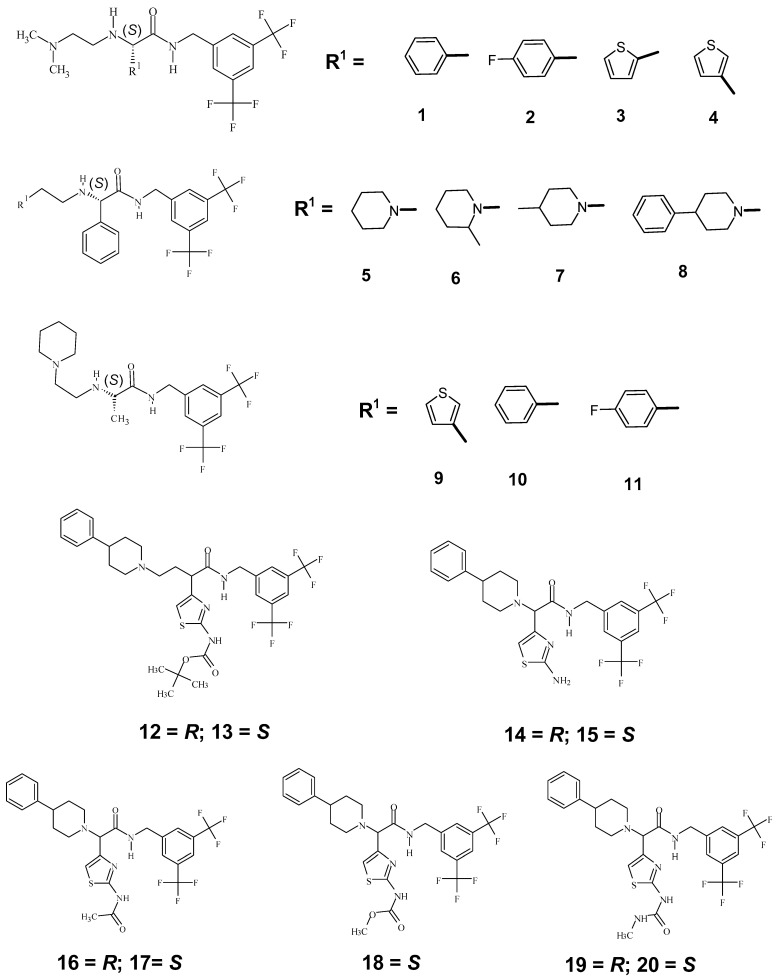
Structures of CCR2 (From 1 to 11, all the compounds are *S* isomers. Among compounds 12 to 20, except 18, the other 8 molecules are 4 pairs of enantiomers. The solid bond indicates the position of attachment of the substituent to the main structure).

The in vitro inhibitory activities of CCR2 antagonists have been reported upon by Merck Laboratories [32,33,34]. Nair et al. [35] selected only 50 molecules that were reported with definite IC_50_ (nM) values for the QSAR studies using chiral sensitive hologram descriptors. All 50 molecules used by Nair et al. [35] are not chiral and only 20 of these are chiral molecules. As the purpose of the current study is to build a QSAR model with chirality descriptors, we used the inhibitory activity, IC_50_ (nM), of the 20 chiral CCR2 antagonists. The selected molecules belong to two structural classes, namely, arylglycinamide and α-aminothiazole-γ-aminobutanoic amide. Among these structural classes, the substituents vary from substituted-phenyl, substituted-thiophenyl, to substituted-piperidinyl. The structures of these molecules are given in Figure 2. The IC_50_ (nM) values are converted to pIC_50_ values by taking −log_10_[IC_50_] (values are given in Table 1).

Nair et al. [35] used hologram descriptors to model the inhibitory activity of CCR2 antagonist activities of 50 compounds comprising 20 chiral compounds. In the hologram analysis, the quality of the model improved only after adding chirality features and the R^2^ value was 0.945 for the 50-molecule data set. As we are using only the 20 chiral molecules, we calculated the R^2^ value for only the 20 molecules as a benchmark to compare the results of our regression analysis. As we used only a subset of 20 molecules, the predicted values using hologram descriptors for this subset of chiral molecules alone were considered, R^2^_pred_ = 0.897.

### 2.2. Calculation of Relative Chirality Indices for R and S Isomers

**Figure 3:**
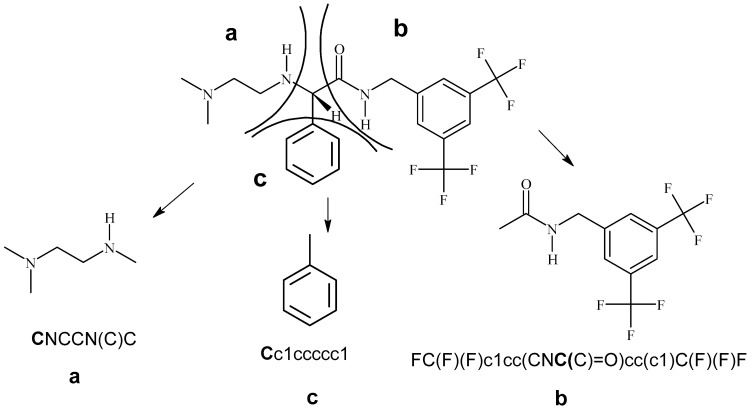
Illustration for application of sequence rule writing of SMILES notation for the substituents (chiral carbon included into the SMILES notation is shown by bold faces).

The steps followed in calculating the relative chirality indices (RCIs) for the *R* and the *S* isomers denoted as *^R^*RCI and *^S^*RCI, respectively, are given below:

1. The four groups attached to the chiral carbon are assigned priority following the Cahn–Ingold–Prelog (CIP) system as a, b, c, and d, where the order of priority is a > b > c > d.
2. SMILES notation is written for each of the four groups and to maintain the connectivity of the atom (vertex) connected to the chiral carbon, the chiral carbon is also included to the groups. This is illustrated for Compound 1, as shown in Figure 3.
3. For each of the four groups (a, b, c, and d), weights are calculated in terms of various topological indices using the SMILES [36,37,38] notations as the input. The topological indices, namely, topo-structural, topo-chemical, triplet, overall connectivity indices, and shape indices ^1^κ and ^2^κ, were calculated as the weights (δ) for each of the four substituents (groups) attached to the chiral carbon as δ_a_, δ_b_, δ_c_, and δ_d_.
4. These weights were then used to compute *^R^*RCI and *^S^*RCI for all the chiral molecules in the data set using Equations (1) and (2).

Invariably, all the twenty molecules have hydrogen (H) as the fourth and the least priority substituent (d) and its δ-value (δ_d_) is zero. If the δ-value of any other substituent computed as its descriptor was zero, then that descriptor was deleted from the list before computing RCIs. The list of various descriptors that could be calculated using the computer programs *POLLY* [39], *INDCAL* [40], and *TRPLET* [41,42] are given in the Supporting Information S1. In addition to the above topological indices, shape indices [43], otherwise called as kappa indices (^1^κ, ^2^κ) of the first order and the second order, were also calculated for the groups. The RCI values on various formalisms are generated for one set of enantiomers by substituting these δ-values in the equations for *^R^*RCI and *^S^*RCI, using Equations (1) and (2), respectively. For the sake of illustration δ_a_, δ_b_, δ_c_, and δ_d_, computed based on only five different formalisms, are shown in Table 2. This could be extended to various orders of the connectivity indices, information contents, and triplet indices.

The RCI values calculated based on the five types of δ-values listed for Compound 1 in Table 2 are given below:

*^R^*RCI^J^ = 52.482; *^S^*RCI^J^ = 51.064

*^R^*RCI^2κ^ = 26.687; *^S^*RCI^2κ^ = 18.683

*^R^*RCI^1χv^ = 64.550; *^S^*RCI^1χv^ = 51.131

*^R^*RCI^IC1^ = 24.112; *^S^*RCI^IC1^ = 18.942

*^R^*RCI^AZV1^ = 26.330; *^S^*RCI^AZV1^ = 19.307

### 2.3. Statistical Analysis

The number of descriptors available are much more than the number of biological responses; therefore, a dimension reduction was applied using principal component analysis (PCA). Before performing the PCA, the descriptors were standardized by a logarithmic transformation, where the transformed descriptor = log_e_(Descriptor + 1). This was necessary because the descriptors differ very much in their dimensions. The PCA and stepwise regression were performed using SPSS 25, while cross validation was carried out using the Statsmodels 0.13.2 module in Python 3.9.13.

## 3. Results and Discussion

Several chirality descriptors based on different theories or concepts of treating molecular connectivity shape attributes were computed. Some of the higher-order connectivity-based descriptors have zero values and the values of higher-order information content become degenerate; therefore, they were removed from the data set. The final set of descriptors contains a lower number of chirality descriptors for each enantiomer (*R* or *S*) than the original number of descriptors computed. The final list of RCIs calculated has 198 different chiral descriptors for each chiral molecule. The number descriptors and the classification according to the formalisms are given in Table 3.

The complete set of chirality descriptors computed for the CCR2 antagonists (20 compounds) is given in the Supporting Information (S1) and the descriptor values are given in the Supporting Information (S2) as an Excel Book.

The intercorrelations of the chirality descriptors are presented in the Supporting Information S2 as one of the Excel worksheets. The RCI with highest correlation with the pI_50_ values is RCI^HIC4^ (r = 0.838) and the least correlated RCI is based on the complementary information content of the zeroth order, RCI^CIC0^ (r = 0.028). Among the chirality descriptors computed, RCIs based on information contents are highly correlated with the bio-efficacy. In the case of connectivity-based chirality descriptors, the cluster and path cluster type of indices are among the top 10 and the overall valence connectivity index-based RCI has the highest negative *r* value of 0.792. The top ten most correlated descriptors from each class of RCI are presented in Table 4.

### 3.1. Regression Analysis

Since the number of descriptors (198) far exceeded the available data points or observations (20), it is necessary to perform a dimension reduction. Principal component analysis (PCA) was used for the dimension reduction and principal components with eigen values > 1 were extracted. A total of six principal components were extracted, and they represented 98.7% of the data variance. The six PCs and their cumulative variance are presented in Table 5. The six principal component scores were used for predicating the inhibitor activity of the chiral CCR2 antagonists.

Regression models using computed molecular descriptors suffer from overfitting, owing to collinearity of predictors. However, when principal component scores (PCSs) are used as predictors, the collinearity problem does not arise because the principal components are orthogonal to each other. Hence, we used the six principal component scores to model the pI_50_ values by stepwise linear regression. The final regression model has only three predictors and the regression equation for the prediction of the inhibitor activity of the chiral CCR2 antagonists is as follows:

(4) $$pI_{50}=7.088+\left(0.728\times PC_{1}\right)+\left(0.469\times PC_{2}\right)+\left(0.3219\times PC_{3}\right)\;n\;=\;20;\;R^{2}=\;0.823;\;\mathrm{Adj}\;R^{2}=\;0.790$$

In the case of a regression model developed with PCS, the utility in future prediction is very limited because for a new molecule, the principal component analysis must be repeated to extract the factor scores. Hence, it is always better to develop prediction models with the actual computed descriptors. In the present case, the relative chirality descriptors that correlate with each of the three principal component scores were selected; if more descriptors have the same correlation, the least degenerate descriptor, i.e., the descriptor that discriminates the chiral molecules effectively, was selected. The three relative chirality descriptors thus selected are RCI*^OPM^*, RCI*^BICO^*, and RCI*^J^*. RCI*^OPM^* is the relative chirality index calculated using the overall path multiplicity of the substituents attached to the chiral center, and similarly, RCI*^BICO^* and RCI*^J^* are the relative chirality descriptors computed using the bond information content of order zero and the Balaban *J* index, respectively. The overall path multiplicity index and Balaban *J* index are related to the branching pattern in the chiral molecule, while the information content on the bond order encodes the bond multiplicity in the molecule. Hence, the branching pattern and the types of bonds in the chiral molecules seem to influence the CCR2 antagonist inhibition of the molecules. However, it should be remembered that correlation is not a proof of causality. The regression equation with the three RCIs is as follows:

(5) $$pI_{50}=\left(0.553\times RCI^{OPM}\right)+(5.763\times RCI^{BIC0}-\left(1.639\times RCI^{J}\right)-7.460\;n=20;\;R^{2}=0.742;\;\mathrm{Adj}\;R^{2}=0.677$$

The benchmark model [35], which is considered the best predictive model, contains four predictors comprising hologram descriptors for atom counts from four to seven atoms, the nature of bonds, the connectivity of atoms, and a chirality parameter. The three-predictor model obtained in this work has molecular descriptors that encode almost identical information about the molecules. Hence, there is good cross talk between the present model and the model developed with hologram descriptors.

A five-fold cross validation was performed, and the results are presented in Table 6. The best R_cv_^2^ of 0.839 was obtained for the second fold. The results of the five-fold cross validation for each fold are given in Supporting Information S3.

### 3.2. Comparison of the Models

**Figure 4:**
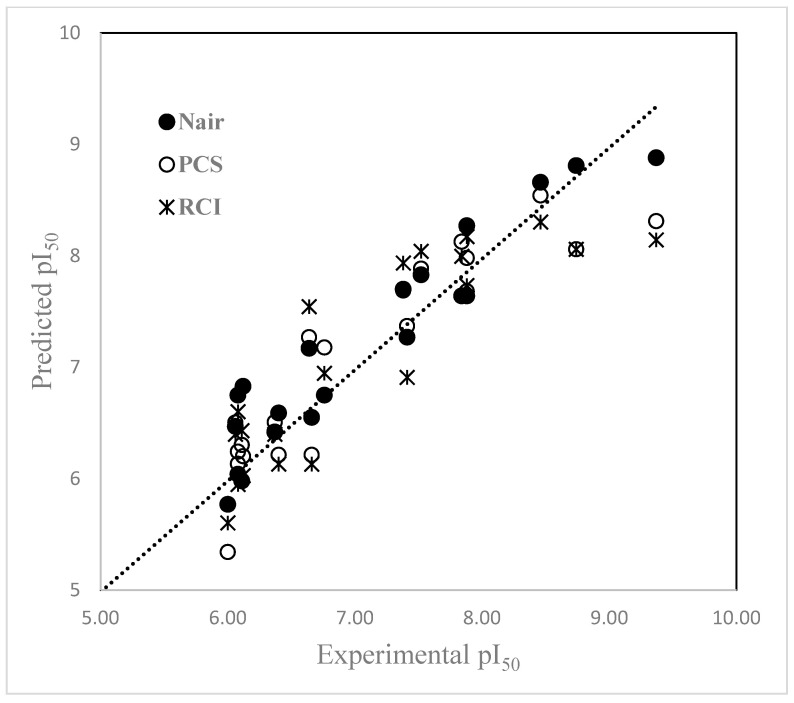
Comparison of predicted pI_50_ vs. actual (experimental) pI_50_ values.

**Figure 5:**
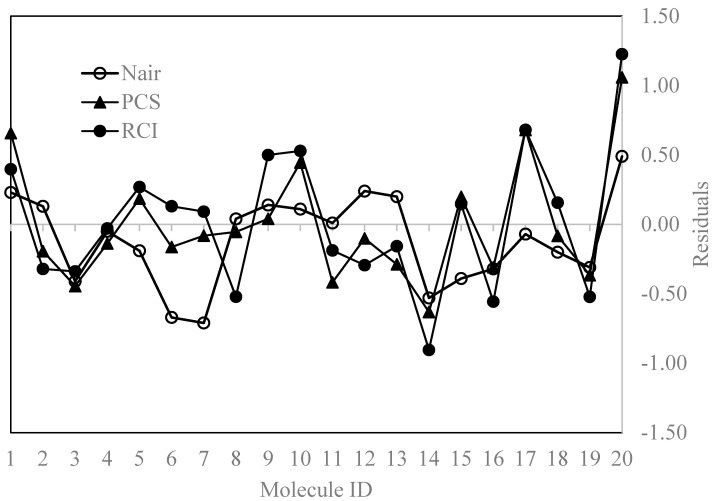
Comparison of residuals of predictions of the various models.

The predicted (pI_50_) values and the residuals of the prediction by each of the models are presented in Table 7 along with that of Nair et al. [35] as the benchmark. It may be noted that the predicted values taken from the literature [35] are part of a set of 50 molecules and the prediction model is not for the 20 chiral molecules only. The plots of the predicted pI_50_ values from each of the regression equations (4) and (5) versus the experimental pI_50_ values are given in Figure 4, and the comparison of residuals of each model is presented in Figure 5. From Figure 4 and Figure 5, it is evident that the predicted pI_50_ values deviate greatly from the experimental values when pI_50_ is > 8.5, indicating that the model did not capture the correct molecular features for molecules with high inhibitory activity. Even in the case of the model developed with hologram descriptors, the predicted value (8.88) for molecule 20 (experimental pI_50_ = 9.37) has a higher deviation from the experimental value. When the pI_50_ value is in the range from 6.0 to 7.0, the predicted values are reasonably closer to the experimental values.

Figure 5 compares the residual for each observation, calculated as the difference between the experimental pI_50_ values and those calculated by the regression models and the benchmark values.

## 4. Conclusions

The results show the application of the relative chirality indices (RCIs) in quantitative chiral structure–activity relationship (QCSAR) modeling. When using RCIs for a given set of chiral molecules, a multidimensional space of chirality descriptors with varying levels of correlations is available. Hence, the RCI approach is a very powerful tool.

I. Because nobody can know a priori what type of interactions will happen between chiral ligands on the one hand and the biotarget on the other, a diverse set of descriptors will have a better chance of success as compared to one single chirality index, no matter how it is derived.
II. The weighting scheme of deriving RCIs is based on calculated properties that have a good physicochemical basis in terms of sigma, pi bonds, count of lone pair, etc., which can characterize many physical and biological properties. For instance, the information content-based RCIs are not only varied, but also take care of neighborhoods of different orders, which are important for properties like the inductive effect, electron donation or withdrawal, etc.

The RCI approach has expanded the descriptor landscape from one single number to a larger set of mutually distinct/uncorrelated indices; thus, a lager net is spread to capture the panorama of ligand–biotarget interactions in diverse situations.

## Figures and Tables

**Table 1:** CCR2 antagonist inhibitory activity pIC_50_.

Molecule ID	pIC_50_ (nM)	Molecule ID	pIC_50_ (nM)
1	6.00	11	6.76
2	6.11	12	7.88
3	6.06	13	7.84
4	6.37	14	6.64
5	6.40	15	7.88
6	6.08	16	7.38
7	6.12	17	8.74
8	6.08	18	8.46
9	7.41	19	7.52
10	6.66	20	9.37

**Table 2:** SMILES notation and group weights δ for the examples shown in Figure 3 (several δ are computed for the group but two samples are shown for each compound).

Sub	SMILES	Group Weights δ_a_, δ_b_, δ_c_, δ_d_				
		J	^2^κ	^1^χ^v^	IC1	AZV1
a	CNCCN(C)C	2.678	2.083	2.564	1.7799	1.422
b	FC(F)(F)c1cc(CNC(C)=O)cc(c1)C(F)(F)F	3.298	3.093	5.027	2.9852	4.180
c	Cc1ccccc1	3.033	1.172	2.411	1.5329	1.711
d	C (d = H)	0	0	0	0	0

Note: J = Balaban index; ^2^κ = shape index; ^1^χ^v^ = first order valence connectivity index; IC1 = dirst order information content; AZV1 = triplet index from adjacency matrix, graph order, and vertex degree; operation y = 1.

**Table 3:** Number of RCIs and their classification.

S. No.	Descriptor Type	Number
1	Triplet indices	100
2	Topo structural indices (TSIs)	24
3	Topochemical indices (TCIs)	20
4	Overall connectivity indices	10
5	Information content (IC)	37
6	ICs for H-suppressed chemical graphs	34
7	Shape indices	2

**Table 4:** Top ten chirality indices (RCIs) highly correlated with CCR2 antagonist affinities.

No.	RCI_Information content_		RCI_Triplet_		RCI_Connectivity_	
	Desc	r	Desc	r	Desc	r
1	HIC4	0.838	AZN4	0.804	V_MPC	−0.792
2	IC2	0.830	DN2S3	−0.801	SC3	0.778
3	HIC2	0.825	DN2S4	−0.801	SPC4	0.776
4	HTIC0	0.825	DN2S5	−0.798	BC3	0.766
5	IC1	0.818	ANZ5	0.789	BPC4	0.765
6	IC0	0.814	ANZ1	0.781	SPC5	0.756
7	HTIC1	0.811	AZV4	0.760	SPC6	0.755
8	HIC3	0.809	ANZ3	0.740	B_OPM	0.741
9	HIC5	0.809	ANN4	0.718	M2	0.739
10	HIORB	0.809	ANN3	0.701	M1	0.738

**Table 5:** Eigen values of principal components and their cumulative variances.

Component	Eigen Values	% of Variance	Cumulative % of Variance
1	153.09	77.3	77.3
2	24.83	12.5	89.9
3	8.79	4.4	94.3
4	4.39	2.2	96.5
5	2.67	1.4	97.9
6	1.71	0.9	98.7

**Table 6:** Results of five-fold cross validation.

Fold No.	Training Set (16 Data)		Test Set (4 Data)
	R^2^	Adj R^2^	R^2^
1	0.743	0.679	0.778
2	0.742	0.677	0.839
3	0.813	0.767	0.512
4	0.798	0.748	0.556
5	0.750	0.688	0.629

**Table 7:** The experimental (actual) and predicted pI_50_ values from different models.

ID	pI_50_ Values				Residuals		
	Exptl	PCS	RCI	Nair [30]	PCS	RCI	Nair
1	6.00	5.34	5.60	5.77	0.66	0.40	0.23
2	6.11	6.30	6.43	5.98	−0.19	−0.32	0.13
3	6.06	6.50	6.40	6.47	−0.44	−0.34	−0.41
4	6.37	6.51	6.40	6.42	−0.14	−0.03	−0.05
5	6.40	6.21	6.13	6.59	0.19	0.27	−0.19
6	6.08	6.24	5.95	6.75	−0.16	0.13	−0.67
7	6.12	6.20	6.03	6.83	−0.08	0.09	−0.71
8	6.08	6.13	6.60	6.04	−0.05	−0.52	0.04
9	7.41	7.37	6.91	7.27	0.04	0.50	0.14
10	6.66	6.21	6.13	6.55	0.45	0.53	0.11
11	6.76	7.18	6.95	6.75	−0.42	−0.19	0.01
12	7.88	7.98	8.17	7.64	−0.10	−0.29	0.24
13	7.84	8.13	8.00	7.64	−0.29	−0.16	0.20
14	6.64	7.27	7.54	7.17	−0.63	−0.90	−0.53
15	7.88	7.68	7.73	8.27	0.20	0.15	−0.39
16	7.38	7.69	7.94	7.70	−0.31	−0.56	−0.32
17	8.74	8.06	8.06	8.81	0.68	0.68	−0.07
18	8.46	8.54	8.30	8.66	−0.08	0.16	−0.20
19	7.52	7.89	8.04	7.83	−0.37	−0.52	−0.31
20	9.37	8.31	8.14	8.88	1.06	1.23	0.49

## Data Availability

Data available upon request to the corresponding author.

## Supplementary Materials

The following supporting information can be downloaded at: https://www.mdpi.com/article/10.3390/molecules30020307/s1, S1: Brief description of molecular descriptors used to compute the weight of the substituents attached to a chiral carbon; S2: Excel workbook: RCI calculated for the 20 chiral CCR2 antagonists and the mutual intercorrelation of RCIs; S3: Results of 5-fold cross validation of each fold.
