# Supplementary material for: Quantitative Structure–Activity Relationship (QSAR) Modeling of Chiral CCR2 Antagonists with a Multidimensional Space of Novel Chirality Descriptors
Source: Molecules. 2025 Jan 14;30(2):307. doi: 10.3390/molecules30020307 (PMC11767949; doi:10.3390/molecules30020307)
Supplement: Supplementary file 1 [file molecules-30-00307-s001.zip › Supplementary materials/CV Results-S3.pdf]

Supporting Information S-3

Results of 5-fold Cross validation for each of the five folds

Explanation of Results

Each page has a separate fold of the CV results. Fold 2 has the best Train and Test R^2 value.
Coef is the coefficient, where const is the intercept value.

0.025 and 0.975 are providing +/- 2 Standard deviations limit of the coefficient values.

FOLD 1:

```
OLS Regression Results
==============================================================================
Dep. Variable:                   PI50   R-squared:                       0.743
Model:                            OLS   Adj. R-squared:                  0.679
Method:                 Least Squares   F-statistic:                     11.56
Date:                Tue, 24 Sep 2024   Prob (F-statistic):           0.000746
Time:                        13:23:56   Log-Likelihood:                -11.602
No. Observations:                  16   AIC:                             31.20
Df Residuals:                      12   BIC:                             34.30
Df Model:                           3
Covariance Type:            nonrobust
==============================================================================
                 coef    std err          t      P>|t|      [0.025      0.975]
------------------------------------------------------------------------------
const         -4.9405      4.833     -1.022      0.327     -15.471       5.590
OPM            0.6079      0.128      4.735      0.000       0.328       0.888
BIC0           7.3377      2.208      3.323      0.006       2.527      12.149
J             -3.3066      2.144     -1.542      0.149      -7.978       1.365
==============================================================================
Omnibus:                        1.530   Durbin-Watson:                   2.119
Prob(Omnibus):                  0.465   Jarque-Bera (JB):                0.565
Skew:                           0.456   Prob(JB):                        0.754
Kurtosis:                       3.130   Cond. No.                         682.
==============================================================================
```

R^2 for Fold 1 Test Data: 0.7776503169384719

FOLD 2: (Best Fold)

OLS Regression Results

Dep. Variable:	PI50	R-squared:	0.742
Model:	OLS	Adj. R-squared:	0.677
Method:	Least Squares	F-statistic:	11.50
Date:	Tue, 24 Sep 2024	Prob (F-statistic):	0.000764
Time:	13:23:56	Log-Likelihood:	-12.525
No. Observations:	16	AIC:	33.05
Df Residuals:	12	BIC:	36.14
Df Model:	3		
Covariance Type:	nonrobust		

	coef	std err	t	P>|t|	[0.025	0.975]
const	-7.4600	5.506	-1.355	0.200	-19.456	4.536
OPM	0.5530	0.111	4.977	0.000	0.311	0.795
BIC0	5.7628	2.594	2.222	0.046	0.111	11.414
J	-1.6390	2.276	-0.720	0.485	-6.599	3.321

Omnibus:	1.166	Durbin-Watson:	2.319
Prob(Omnibus):	0.558	Jarque-Bera (JB):	0.634
Skew:	0.479	Prob(JB):	0.728
Kurtosis:	2.820	Cond. No.	740.

R^2 for Fold 2 Test Data: 0.8386515868913238

FOLD 3:

OLS Regression Results

Dep. Variable:	PI50	R-squared:	0.813
Model:	OLS	Adj. R-squared:	0.767
Method:	Least Squares	F-statistic:	17.44
Date:	Tue, 24 Sep 2024	Prob (F-statistic):	0.000113
Time:	13:23:56	Log-Likelihood:	-8.9978
No. Observations:	16	AIC:	26.00
Df Residuals:	12	BIC:	29.09
Df Model:	3		
Covariance Type:	nonrobust		

	coef	std err	t	P>|t|	[0.025	0.975]
const	-6.0821	3.935	-1.546	0.148	-14.655	2.491
OPM	0.5314	0.087	6.093	0.000	0.341	0.721
BIC0	6.7599	1.644	4.111	0.001	3.178	10.342
J	-2.3464	1.422	-1.650	0.125	-5.445	0.752

Omnibus:	7.482	Durbin-Watson:	1.535
Prob(Omnibus):	0.024	Jarque-Bera (JB):	4.244
Skew:	1.050	Prob(JB):	0.120
Kurtosis:	4.399	Cond. No.	632.

R^2 for Fold 3 Test Data: 0.5123392775517821

FOLD 4:

```
                            OLS Regression Results
==============================================================================
Dep. Variable:                   PI50   R-squared:                       0.798
Model:                            OLS   Adj. R-squared:                  0.748
Method:                 Least Squares   F-statistic:                     15.81
Date:                Tue, 24 Sep 2024   Prob (F-statistic):           0.000181
Time:                        13:23:56   Log-Likelihood:                -8.2427
No. Observations:                  16   AIC:                             24.49
Df Residuals:                      12   BIC:                             27.58
Df Model:                           3
Covariance Type:            nonrobust
==============================================================================
                 coef    std err          t      P>|t|      [0.025      0.975]
------------------------------------------------------------------------------
const         -5.5841      4.283     -1.304      0.217     -14.916       3.747
OPM            0.5045      0.080      6.330      0.000       0.331       0.678
BIC0           6.1963      1.557      3.980      0.002       2.804       9.589
J             -2.1269      1.411     -1.507      0.158      -5.201       0.948
==============================================================================
Omnibus:                        0.366   Durbin-Watson:                   2.380
Prob(Omnibus):                  0.833   Jarque-Bera (JB):                0.365
Skew:                           0.291   Prob(JB):                        0.833
Kurtosis:                       2.543   Cond. No.                         717.
==============================================================================
```

R^2 for Fold 4 Test Data: 0.5563971790580429

FOLD 5:

OLS Regression Results

Dep. Variable:	PI50	R-squared:	0.750
Model:	OLS	Adj. R-squared:	0.688
Method:	Least Squares	F-statistic:	12.03
Date:	Tue, 24 Sep 2024	Prob (F-statistic):	0.000627
Time:	13:23:56	Log-Likelihood:	-12.002
No. Observations:	16	AIC:	32.00
Df Residuals:	12	BIC:	35.09
Df Model:	3		
Covariance Type:	nonrobust		

	coef	std err	t	P>|t|	[0.025	0.975]
const	-2.7838	5.401	-0.515	0.616	-14.552	8.985
OPM	0.5504	0.096	5.742	0.000	0.342	0.759
BIC0	5.5802	2.421	2.305	0.040	0.305	10.855
J	-2.7369	1.966	-1.392	0.189	-7.020	1.546

Omnibus:	3.080	Durbin-Watson:	1.789
Prob(Omnibus):	0.214	Jarque-Bera (JB):	1.332
Skew:	0.671	Prob(JB):	0.514
Kurtosis:	3.446	Cond. No.	722.

R^2 for Fold 5 Test Data: 0.6294473219045892
