# Supplementary material for: Quantitative Structure–Activity Relationship (QSAR) Modeling of Chiral CCR2 Antagonists with a Multidimensional Space of Novel Chirality Descriptors
Source: Molecules. 2025 Jan 14;30(2):307. doi: 10.3390/molecules30020307 (PMC11767949; doi:10.3390/molecules30020307)
Supplement: Supplementary file 1 [file molecules-30-00307-s001.zip › Supplementary materials/Molecular descriptors for computing RCIs-S1.pdf]

Supplementary Information-1

List of molecular descriptors computed to assign weights (δ) for the substituents attached the chiral center (carbon).

If Wiener index W of each of the four substituents is used as the weight (δ), then the relative chirality index for *R*-isomer will be denoted as $^{R}RCI^{W}$ and that for the *S*-isomer will be denoted as $^{S}RCI^{W}$. Similarly, when Balaban index J is used for computing the weights of the substituents then the pair of RCIs is $^{R}RCI^{J}$ and $^{S}RCI^{J}$.

Graph theoretical descriptors

$\mathbf{I^{W}_{D}}$	Information index for the magnitudes of distances between all possible pairs of vertices of a graph
$\mathbf{I_h{}^{W}{}_{D}}$	Mean information index for the magnitude of distance
**W**	Wiener index = half-sum of the off-diagonal elements of the distance matrix of a graph
**H**	Harary index = half-sum of reciprocal square of the elements of the distance matrix of a graph
**J**	Balaban index = sum of the reciprocal square root of degree of all adjacent pairs
$\mathbf{I_C{}^{D}}$	Information content of the distance matrix
**IC**	Information content of the distance matrix partitioned by frequency of occurrences of distance
**O**	Order of neighborhoods when ICr reaches its maximum value for the hydrogen-filled graph
**M1**	Zagreb group parameter = sum of square of degree over all vertices
**M2**	Zagreb group parameter = sum of cross-product of degrees over all neighboring (connected) vertices M2

Topo-structural descriptors

$^{h}\chi$	Path connectivity index of order *h* = 0-6
$^{h}\chi_{c}$	Cluster connectivity index of order *h*) 3-6
$^{h}\chi_{ch}$	Chain connectivity index of order *h*) 3-6
$^{h}\chi_{c}$	Path-cluster connectivity index of order *h*) 4-6
P_h	Number of paths of length *h* = 0-10

Topo-chemical descriptors (bond-order connectivity)

$^{h}\chi^{b}$	Bond path connectivity index of order *h* = 0-9
$^{h}\chi_{c}{}^{b}$	Bond cluster connectivity index of order *h* = 3-6
$^{h}\chi_{ch}{}^{b}$	Bond chain connectivity index of order *h* = 3-6
$^{h}\chi_{Pc}{}^{b}$	Bond path-cluster connectivity index of order *h* = 4-6

Topo-chemical descriptors (valence connectivity)

$^{h}\chi^{v}$	Valence path connectivity index of order h = 0-9
$^{h}\chi_{c}^{v}$	Valence cluster connectivity index of order h = 3-6
$^{h}\chi_{ch}^{v}$	Valence chain connectivity index of order h = 3-6
$^{h}\chi_{Pc}^{v}$	Valence path-cluster connectivity index of order h = 4-6

Information theoretic descriptors

**IC_r**	Mean information content or complexity of a graph based on the rth (r = 0-6) order neighborhood of vertices in a hydrogen-filled graph
**SIC_r**	Structural information content for r^{th} (r = 0-6) order neighborhood of vertices in a hydrogen-filled graph
**CIC_r**	Complementary information content for rth (r = 0-6) order neighborhood of vertices in a hydrogen-filled graph
**TIC_r**	Total information content for r^{th} (r = 0-6) order neighborhood of vertices in a hydrogen-filled graph
**BIC_r**	Bond information content for r^{th} (r = 0-6) order neighborhood of vertices in a hydrogen-filled graph

Information theoretic descriptors for hydrogen suppressed graphs

**HIC_r**	Mean information content or complexity of a hydrogen suppressed graph based on the rth (r = 0-6) order neighborhood of vertices in a hydrogen suppressed graph
**$HSIC_r$**	Structural information content for r^{th} (r = 0-6) order neighborhood of vertices in a hydrogen suppressed graph
**$HCIC_r$**	Complementary information content for rth (r = 0-6) order neighborhood of vertices in a hydrogen suppressed graph
**$HTIC_r$**	Total information content for r^{th} (r = 0-6) order neighborhood of vertices in a hydrogen suppressed graph
**$HBIC_r$**	Bond information content for r^{th} (r = 0-6) order neighborhood of vertices in a hydrogen suppressed graph
**I_{ORB}**	Information content or complexity of the hydrogen suppressed graph at its maximum neighborhood of vertices
**O_{ORB}**	Order of neighborhoods when ICr reaches its maximum value for the hydrogen suppressed graph

Overall connectivity indices

**SumR**	Sum of connectivity indices of all orders ($\Sigma\, ^{h}\chi$)
**VSumR**	Sum of valence connectivity indices of all orders ($\Sigma\, ^{h}\chi^{v}$)
**BSumR**	Sum of bond order connectivity indices of all orders ($\Sigma\, ^{h}\chi^{b}$)
**OPM**	Overall path multiplicity $\Sigma\, ^{h}\chi \times P_h$
**VOPM**	Overall path multiplicity of valence connectivity $\Sigma\, ^{h}\chi^{v} \times P_h$

BOPM Overall path multiplicity of bond order connectivity $\Sigma\, {}^h\chi^b \times P_h$

MPC Mean path connectivity index $\Sigma\, {}^h\chi \div P_h$

VMPC Mean path connectivity index based on valence connectivity $\Sigma\, {}^h\chi^v \div P_h$

BMPC Mean path connectivity index based on bond connectivity $\Sigma\, {}^h\chi^b \div P_h$

SumK Sum of all paths of length 1 to h ΣP_h

Triplet indices – Topo-structural (connectivity)

DN^2S_y Triplet index from distance matrix, square of graph order, and distance sum; operation y=1–5

DN_21_y Triplet index from distance matrix, square of graph order, and number 1; operation y=1–5

$AS1_y$ Triplet index from adjacency matrix, distance sum, and number 1; operation y=1–5

$DS1_y$ Triplet index from distance matrix, distance sum, and number 1; operation y=1–5

ASN_y Triplet index from adjacency matrix, distance sum, and graph order; operation y=1–5

DSN_y Triplet index from distance matrix, distance sum, and graph order; operation y=1–5

DN^2N_y Triplet index from distance matrix, square of graph order, and graph order; operation y=1–5

ANSy Triplet index from adjacency matrix, graph order, and distance sum; operation y=1–5

$AN1_y$ Triplet index from adjacency matrix, graph order, and number 1; operation y=1–5

ANN_y Triplet index from adjacency matrix, graph order, and graph order again; operation y=1–5

ASV_y Triplet index from adjacency matrix, distance sum, and vertex degree; operation y=1–5

DSV_y Triplet index from distance matrix, distance sum, and vertex degree; operation y=1–5

ANV_y Triplet index from adjacency matrix, graph order, and vertex degree; operation y=1–5

Triplet Indices - Topochemical

AZV_y Triplet index from adjacency matrix, atomic number, and vertex degree; operation y=1–5

AZS_y Triplet index from adjacency matrix, atomic number, and distance sum; operation y=1–5

ASZ_y Triplet index from adjacency matrix, distance sum, and atomic number; operation y=1–5

AZN_y Triplet index from adjacency matrix, atomic number, and graph order; operation y=1–5

ANZ_y Triplet index from adjacency matrix, graph order, and atomic number; operation y=1–5

DSZ_y Triplet index from distance matrix, distance sum, and atomic number; operation y=1–5

DN^2Z_y Triplet index from distance matrix, square of graph order, and atomic number; operation y=1–5
